# Prognostic Value of the Number of Circulating Tumor Cells in Patients with Metastatic Non-Small Cell Lung Cancer

**DOI:** 10.3390/mi16040470

**Published:** 2025-04-15

**Authors:** Arthur B. Volovetsky, Victoria A. Novikova, Anastasia Boloban, Aleksej S. Rzhevskiy, Alina Kapitannikova, Elena G. Ovchinnikova, Tatjana P. Klejmentjeva, Vladislav A. Grishin, Yana Pigareva, Andrei V. Zvyagin, Majid Ebrahimi Warkiani, Anna V. Maslennikova

**Affiliations:** 1 Institute for Regenerative Medicine, Sechenov University, Moscow 119991, Russia; voloveckiy91@gmail.com; 2 Central Research Laboratory, Privolzhsky Research Medical University, Nizhny Novgorod 603005, Russia; 3 Nizhny Novgorod Research Institute of Clinical Oncology, Nizhny Novgorod 603163, Russia; 4 Neurotechnology Department, Lobachevsky State University of Nizhny Novgorod, Nizhny Novgorod 603950, Russia; 5 Institute of Molecular Theranostics, Sechenov University, Moscow 119991, Russia; rzhevskiy01@gmail.com (A.S.R.); kapitannikova_a_yu@staff.sechenov.ru (A.K.); 6 Australian Research Council Centre of Excellence for Nanoscale Biophotonics, Macquarie University, Sydney, NSW 2109, Australia; andrei.zvyagin@mq.edu.au; 7 Research Center for Translational Medicine, Sirius University of Science and Technology, Sochi 354340, Russia; 8 School of Biomedical Engineering, University of Technology Sydney, Sydney, NSW 2007, Australia

**Keywords:** circulating tumor cells, non-small cell lung cancer, metastases, chemotherapy, targeted therapy, microfluidic, CTCs detection

## Abstract

Investigating the molecular and genetic characteristics of circulating tumor cells (CTCs) presents a promising approach for personalizing treatment in patients with malignant neoplasms, given the limitations of traditional biopsy and histopathology. This study aimed to isolate, characterize, and analyze CTC dynamics in the peripheral blood of 30 patients with metastatic lung cancer to develop criteria for treatment response and prognosis. We detected CTCs before the start of the treatment and monitored changes during treatment, correlating these with responses evaluated by standard imaging methods. A decrease in the CTCs in the course of the therapy was linked to a favorable tumor response, while the stable CTC counts indicated a lack of response and poor survival prognosis. The OS of patients was analyzed and compared with the initial number of CTCs in peripheral blood samples. The significant reductions in median OS were evident in patients with >3 total CTCs at baseline compared to those with ≤3 total CTCs (median survival 26 months, *n* = 10, vs. median survival 8 months, *n* = 19, respectively with HR = 2.6, 95% CI 1.07 to 6.4).

## 1. Introduction

Circulating tumor cells (CTCs) were first described in 1869 by Australian physician Thomas Ashworth, who discovered them in the peripheral blood of a patient who died of cancer (Ashworth 1869). CTCs as biomarkers for the diagnosis and prognosis of cancer began to be used only in the last 10–15 years, with the development and introduction of methods for their separation into clinical practice [1].

Currently, the prognosis and monitoring of the effectiveness of cancer patient treatment is based on clinical and instrumental data in accordance with the RECIST score, which can be detected significantly later compared to the actual onset of progression. In this situation, an attractive alternative is the use of biomarkers that can be detected in peripheral blood, particularly CTCs. CTCs are one of the most promising objects for studying the heterogeneity of tumors and the process of metastasis [2,3,4]. Identification of CTCs in the peripheral blood of patients correlates with the lowest overall survival (OS) in a cohort of patients with pancreatic cancer [5], ovarian cancer [6], metastatic prostate cancer [7,8], breast cancer [9,10], colon [11], bladder [12], and gastric cancer [13].

Lung cancer accounts for 12.9% of all new cancer cases globally [14] and includes a diverse range of malignant epithelial tumors with varying origins, histological structures, clinical courses, and treatment outcomes. The efficacy and prognostic value of circulating tumor cells (CTCs) in non-small cell lung cancer (NSCLC) are controversial based on the existing research. Some studies devoted to this problem concerned the assessment of minimal residual disease after surgical treatment [15,16,17].

A prognostic significance of CTC amount in peripheral blood was investigated in the studies by Krebs et al., and Lindsay et al. [18,19,20]. According to these studies, ≥5 CTCs in 7.5 mL of peripheral blood were selected as a poor prognostic criterion. In the study by Tong et al. [21] poor criteria for survival were indicated as ≥8 CTCs in 7.5 mL before treatment and an increment in the number of CTCs post-treatment. In the study by Zhou at el. an increased number of CTCs was indicated either before and after the chemotherapy in case of poor response to chemotherapy [22]. In Zhang at el. [23] baseline CTC count was found to be an independent prognostic factor for overall and disease-free survival. Furthermore, no correlation between CTC count and changes in tumor size after chemotherapy was indicated.

The number of CTCs before treatment appears to be important as a prognostic factor in patients with locally advanced lung cancer with a cut-off of 1 CTC/7.5 mL blood during both standard chemotherapy and targeted therapy with tyrosine kinase inhibitors [24].

Most of the presented works indicate the positive prognostic value of a decrease in the number of CTCs after the start of drug treatment [25], however, there is data that changes in CTC counts after the first course of drug therapy were linked to patient survival, with higher survival rates in patients whose CTC counts increased during the initial three months, possibly reflecting the impact of chemotherapy on tumor tissue [26].

Thus, previous studies have demonstrated that the presence of CTCs appeared to be a universal criterion for unfavorable prognosis in patients with locally advanced and metastatic lung cancer [27] and the negative prognostic value of their presence in peripheral blood is currently beyond doubt. However, the cut-off level for the number of CTCs and also the influence of molecular genetic characteristics of the tumor (presence of driver mutation) that is prognostic for patient survival remains unclear.

One of the problems encountered when isolating CTCs from the blood of patients with lung cancer is the lack of a uniform testing technique. Positive immunoselection employs antibodies against surface adhesion molecules, such as epithelial cell adhesion molecule (EpCAM), to capture CTCs. The use of these techniques makes it possible to isolate CTCs from a significantly smaller proportion of patients compared to EpCAM-independent assays [28], based on the physical peculiarities of CTCs, such as size [29,30,31], density [32] and electrical charge of the cells [33]. Microfluidic systems provide a wide range of studies with minimal sample volumes and the ability to study biological processes dynamically [34,35]. Another advantage is the ability to separate tumor cells regardless of their immunophenotype, i.e., at any location of the tumor. In our study, a microfluidic system was used to isolate CTCs from peripheral blood in patients with metastatic non-small cell lung cancer (NSCLC) to monitor the disease course and estimate the prognosis of the disease.

## 2. Materials and Methods

### 2.1. Patient Information and Study Design

Thirty patients with metastatic NSCLC were included in the study (Table 1). Each patient signed a written informed consent before data collection. Patients with clinical and histological (biopsy or postoperative material) verification of the primary tumor and metastatic foci (except for bone metastases) were eligible to enter the study. The metastatic foci were verified by using the protocol of intravital pathological examination and instrumental examination data (CT of the chest, MRI of the abdominal cavity/brain, bone SPECT/CT). The inclusion criteria were:

- Histologically proven primary diagnosed non-small cell metastatic lung cancer (IV st.);
- Histologically proven NSCLC of any stage after previous treatment with newly diagnosed distant metastases;
- No medical treatment for metastatic cancer;
- ECOG performance status 0–2;
- Satisfactory hematological parameters (Hb more than 9 g/L, neutrophils more than 3 × 10^9^, platelets more than 100 × 10^9^)
- Satisfactory liver function.

Exclusion criteria were a functional status on the ECOG scale > 2 and discontinuation of the drug therapy due to exhaustion of therapeutic options. One patient who died 2.5 months after the first blood sample collection from the cancer-independent reason was also excluded from the analysis.

Peripheral blood samples were collected from all patients 1 day before the start of the treatment for metastatic lung cancer, then after every two courses (chemotherapy, immunotherapy, targeted therapy) before every third course. The monitoring was carried out for 12 months from the start of the treatment. A maximum number of the blood samples was 6. In one patient, the sample was obtained only once before the start of the treatment. Number of CTCs in the peripheral blood samples was determined and correlated with the clinical course of the disease and the results of the instrumental studies.

### 2.2. Method of Collecting Peripheral Blood and Separating CTCs

Patient peripheral blood samples were collected from the cubital vein into disposable sterile vacuum tubes containing K3-EDTA anticoagulants and processed within 3 h. To prevent epithelial cells from entering the sample after skin puncture, the first milliliter of the patient’s blood was utilized, and then 9 mL of blood was collected. The blood was leased using room temperature lysis buffer (MACS^®^ Separation Buffer, Miltenyi Biotec, Bergisch Gladbach, Germany) for 20 min by constantly stirring at a blood:buffer ratio of 1:2.5 to remove red blood cells. Next, the blood lysate was centrifuged (ELMI CM-6M 5 min, 500× *g*), the supernatant was discarded, and the sediment containing the target CTCs was diluted with phosphate-buffered saline (PBS) to 10 mL.

The resulting cell suspension was filled in a 10 mL syringe and pumped through the device using a syringe pump (SpLab01, DK Infusetek Co., Ltd., Shanghai, China) connected to the microchannel of a spiral microfluidic chip (width of the channel—600 μm, the inner/outer heights—80 and 130 μm, respectively) [34,36] through a plastic tube (diameter 0.5 mm). The flow rate was set to 1.7 mL/min for all experiments.

**Figure 1:**
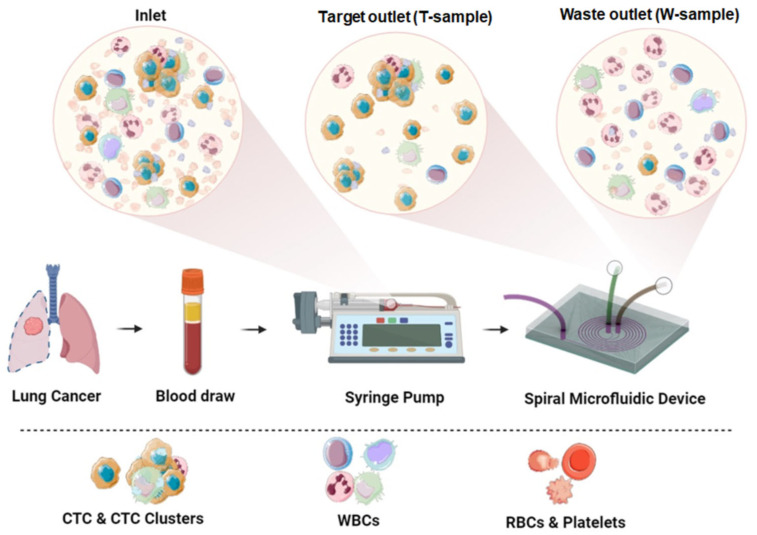
Experimental scheme. Prepared patient peripheral blood sample was filled in a syringe and pumped through the microfluidic device using a syringe pump. Inertial cell separation occurs in the spiral channel: CTCs are collected in a Target tube (T-sample), blood cells are collected in a Waste tube (W-sample).

In the microchannel, cells were separated based on their size and mechanical compliance using inertial focusing of the cells in the liquid due to a combination of inertial and Dean forces. The CTCs were focused near the inner wall of the channel at the Target tube (T-sample), while most of the blood cells were focused near the outer channel at the Waste tube (W-sample). The functionality of the chip was also based on the rectangular-trapezoidal cross-section of the microfluidic channel (Figure 1).

### 2.3. Method for Characterizing Isolated CTCs Using a Panel of Fluorescent Antibodies

The cells isolated from the blood were characterized to confirm their epithelial origin. The target fraction was transferred to a solution of 4% paraformaldehyde, the original tube was washed with this solution and incubated for 10 min. After incubation, the sample was centrifuged using a centrifuge (ELMI CM-6M, Elmi, Saint-Petersburg, Russia) at 500× *g* for 5 min. The supernatant was transferred to the drain, taking maximum care to avoid damaging the sediment. Phosphate buffer at the amount of 25 mL was added to the sediment, resuspended, and immediately centrifuged at 500× *g* for 5 min. The supernatant was carefully transferred to the drain without damaging the sediment. The pellet was resuspended in 400 μL of PBS. The assembled cassettes were installed in a cytocentrifuge (Domel Awel C12, Domel, Železniki, Slovenia). The finished glasses with the applied cell suspension were placed in 0.2% PBST for 5 min to permeabilize the cell membrane. Next, the slides with fixed and permeabilized cells were placed in a block medium (5% serum albumin solution in 0.05% PBST) for 1 h at room temperature.

Primary staining was performed with fluorescent antibodies Anti-pan Cytokeratin antibody [PCK-26] (Abcam) for 2 h. After incubation, the slides were washed passively in PBS solution for 8 min. Secondary staining was carried out with fluorescent antibodies Goat Anti-Mouse IgG H&L (Alexa Fluor^®^ 488) (Abcam Inc., Waltham, MA, USA) (concentration 1 μg/mL in 1% serum albumin solution in 0.05% PBST) according to the above scheme for 1 h, then preparations were washed passively in PBS solution for 8 min. Next, stained and washed slides were placed again in the block medium for 30 min. Primary staining was carried out with fluorescent antibodies Rabbit Anti-CD45 antibody (Abcam) (concentration 1 μg/mL in 1% serum albumin solution in 0.05% PBST) for 2 h, then the preparation was passively washed in PBS solution for 8 min. Secondary staining was performed with fluorescent antibodies Goat Anti-Rabbit IgG H&L (AlexaFluor^®^ 594) (Abcam Inc., Waltham, MA, USA) (concentration 1 μg/mL) for 1 h, then the slides were washed passively in PBS solution for 8 min. After brief air-drying, 8 μL of mounting medium containing DAPI (Abcam) was applied to the area with cells, followed by the area covered with a coverslip. The edges of the coverslip were sealed using a clear varnish.

Fluorescence and photographic images were obtained using an Axio Observer Z1 LSM 710 DUO confocal laser scanning microscope (Carl Zeiss, Oberkochen, Germany). CTCs were defined as PCK-26-positive and CD45-negative cells and detected and counted manually by using the microscope. Areas with detected CTCs were captured by imaging. When counting cells in a sample, non-nucleated cells (DAPI-negative) with cytoplasm stained with cytokeratin (PCK-positive) were considered as an artifact and excluded from the count. All PCK-positive/DAPI-positive cells were counted as CTCs regardless of the size of the nucleus, since it was assumed that the nucleus may be partially “lost” during sample processing.

In tumors with identified driver mutations, confirmation of the tumor nature of CTCs was carried out using digital droplet PCR (QX200™ Droplet Digital PCR, Bio-Rad, Hercules, CA, USA).

### 2.4. Statistical Analysis

The survival was evaluated depending on the cutoff level. In our study, the criterion of one-year survival was chosen as the criterion of an unfavorable prognosis. Patients who survived less than one year were assigned to the unfavorable group, and those who survived more than one year were assigned to the favorable group. The cutoff level was chosen as the median of CTC count at the baseline in the favorable group and was 3 CTCs.

The data were reported as median ± median absolute deviation and percentages. The correlation between CTCs count and lifespan was performed by Spearman Rank Order Correlation test.

Survival curves were plotted using the Kaplan–Meier analysis and the log–rank test was used to determine the statistical differences between life curves. Overall survival (OS) was measured from date of baseline blood sample to date of death or was censored at last follow-up. Associations between CTC counts with OS were estimated using hazard ratios (HR) and 95% confidence intervals (CI), calculated by univariate and Cox proportional hazards models.

The Mann-Whitney Rank Sum Test was performed to compare the CTC number in 2 groups. Statistical significance was fixed at *p*-values less than 0.05. Statistical analysis was performed using the SigmaPlot 12.5.

## 3. Results

### 3.1. Patient Characteristics

All patients were older than 41 years. The diagnosis were adenocarcinoma (70%, *n* = 21), followed by squamous cell carcinoma (30%, *n* = 9). 70% of the patients (*n* = 21) initially had Stage IV, 23.3% (*n* = 7), Stage III, and 6.7% (*n* = 2) Stage I and II of the disease. Mutations in the EGFR and ALK genes were identified in 9 tumors (30%). The data of the study cohort are shown in Table 1.

### 3.2. Patient Survival

The date of the start of drug therapy was taken as the beginning of the observation. A follow-up period for patients ranged from 2 to 41 months, with a median follow-up period of 18 months. At the time of analysis, 26 patients had died within a period from 2 to 29 months—25 from the disease progression, and 1 patient died from cancer-unrelated cause (protocol code 11). 4 patients were alive receiving treatment. The one-year survival rate was 43.3%, the three-year survival rate—13.3% and the median life expectancy was 10.5 months.

### 3.3. CTCs Detection

**Figure 2:**
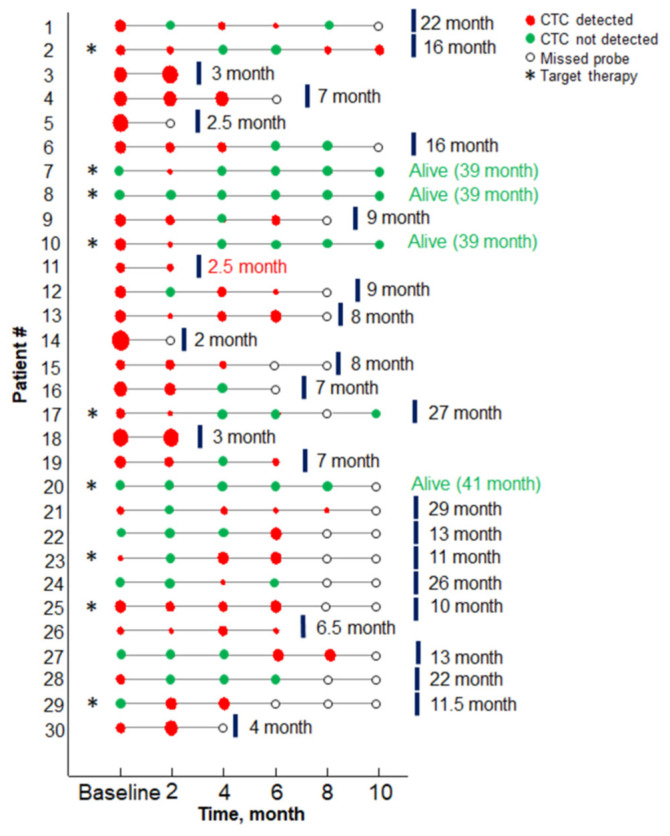
Results of the CTCs assay results versus treatment timing for each patient. Red circles indicate the probe containing CTCs, size encodes the number of CTC (minimal, maximal sizes—1, 16 CTCs, respectively). Green circles correspond to probes with no CTC detected. Patients with the driver mutation are marked with an asterisk. Red text marks the patient case, when death occurred from the unrelated disease (protocol code 11).

CTCs were detected in 23 (76.7%) of NSCLC cancer patients before the start of the treatment (baseline) and in 28 out of 30 patients during the study. CTCs count ranged from 0 to 16 per 9 mL of the blood at the baseline, with a median CTC value of 5.0 ± 3.4 per 9 mL/blood. The initial number of CTCs did not depend on the histological structure of the tumor and metastatic site. Evolution of the CTC numbers during the treatment in 30 patients included in the study, as well as their disease outcomes, are presented in Figure 2 and Table S1.

### 3.4. CTCs Count Associated with Overall Survival

The peripheral CTC count in the baseline had a negative correlation with the lifespan (Correlation Coefficient = −0.6).

**Figure 3:**
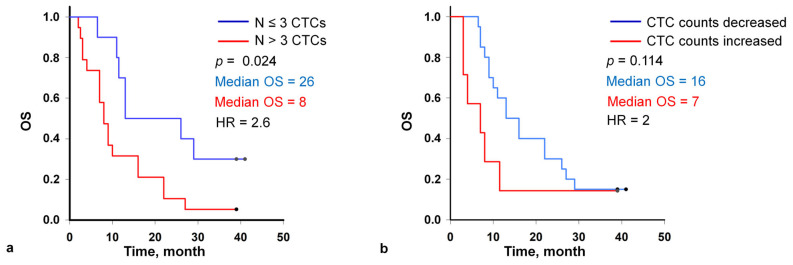
Kaplan-Meier curves for overall survival of patients with the fewer than 3 and ≥3 circulating tumor cells (CTCs) per 9 mL of blood at the baseline (**a**), with decreasing and increasing CTC count in the second sample (2 months after the start of the therapy) (**b**).

OS of patients was analyzed and compared with the initial number of CTCs in the peripheral blood samples (Figure 3a). After a median follow-up of 41 months, univariate analyses showed significant reductions in the median OS evident in patients with >3 total CTCs at the baseline as compared to those with ≤3 total CTCs (median survival 26 months, *n* = 10, vs. median survival 8 months, *n* = 19, respectively with HR = 2.6, 95% CI 1.07 to 6.4). The one-year survival in patients with >3 total CTCs at baseline was 31%, a three-year—3%. In patients with ≤3 total CTCs the one-year and three-year survival was 70% and 30%, respectively (*p* = 0.024).

The second blood samples for CTC analysis were obtained from 27 patients after two cycles of chemotherapy from the baseline. Considering the first and second time points together, the CTC count increased in 7 patients and decreased or remained unchanged in 20 patients, including 0 counts (Figure 3b). Patients with the decreased CTC count exhibited the longer median survival (16 vs. 7 months; log-rank test *p* = 0.114; HR, 2; 95% CI 0.8 to 5.3) compared with patients with the increased CTC count while the statistical significance was not established.

### 3.5. Target Therapy

Of the 30 patients included in the study, driver mutations in the EGFR and ALK genes were detected in lung tumors in 9 patients, which were administered to targeted therapy. Patients with mutations in the EGFR gene received treatment with afatinib and osemertinib, 1 patient with a mutation in the ALK gene was treated with alectinib. Targeted therapy allowed significantly extending the life of 4 patients who were alive at the moment of analysis. The initial number of CTCs in this group varied from 0 to 8. Survival analysis shows that the median survival was 27 months, one-year survival was 66%, three-year survival was 44%, with significant differences compared to patients with tumors without identified mutations (median survival was 9 months; Mann-Whitney Rank Sum Test, *p* < 0.005).

### 3.6. Clinical Example

#### 3.6.1. A Clinical Example Demonstrating a Favorable Outcome of the Disease and the Evolution of the Number of CTCs Detected in the Peripheral Blood

**Figure 4:**
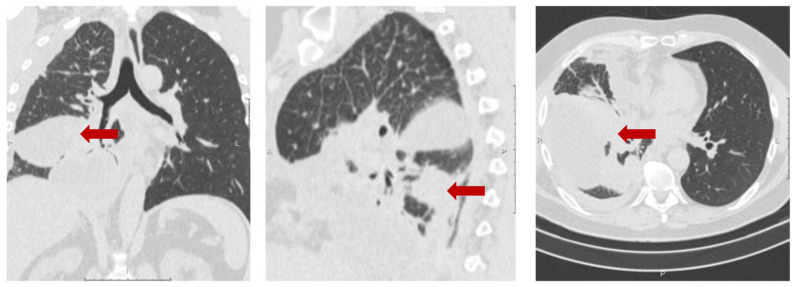
Protocol code 10: CT scan of the chest with intravenous contrast enhancement from 17 December 2020 (before the start of treatment): mass formation of the low lobe of the right lung with pre-disintegration (55 × 38 mm, along the periphery with pleural effusion 103 × 38 mm) involving the right lower lobe, intermediate bronchus and middle lobe bronchus (indicated by arrow), single small foci of the right lung up to 4 mm (mts), hydrothorax on the right with a tendency to encystment.

The 53-year-old male patient (protocol code 10). He considered himself sick in May 2020, when a periodic right side chest pain appeared. In July 2020, he was diagnosed with right-sided lower lobe pneumonia, for which he was treated in a therapeutic hospital without an effect. A CT scan of the chest revealed a tumor of the right lower lobe bronchus, atelectasis of the lower lobe of the right lung, right-sided massive hydrothorax, and emphysema (Figure 4). Based on the results of the examination (CT scan of the chest, fibrobronchoscopy with biopsy, pleural puncture, video thoracoscopy with biopsy), he was diagnosed with a cancer of the right lower lobe bronchus T4N2aM1a, dissemination along the pleura. Histological examination No. 5503/1 dated 12 February 2020: G2 adenocarcinoma. Genetic testing of tumor tissue revealed a mutation in the ALK gene.

**Figure 5:**
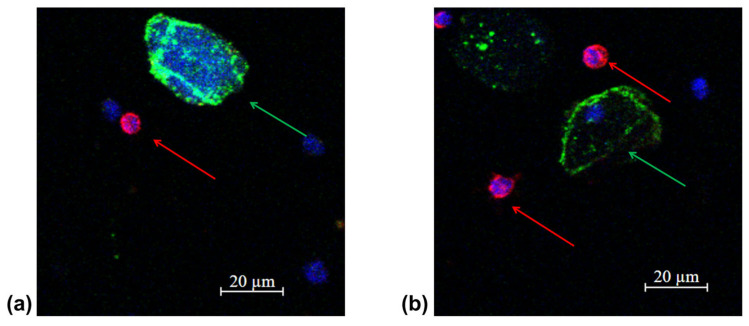
Representative fluorescence confocal microscopy images of tumor cells isolated from the patient’s peripheral blood sample (protocol code 10) at the baseline (**a**) and after 3 treatment courses (**b**). CTC are marked by green arrows, anuclear PCK-positive structure is an artifact marked by white arrow, white blood cells by red arrows in the target sample labelled with CD45 (Red); PCK-26 (Green); DAPI (Blue).

In accordance with the clinical recommendations of the Russian Association of Oncologists (AOR), taking into account the molecular profile of the tumor (ALK mutation), targeted therapy with the drug Alecensa (alectinib, F. Hoffmann-La Roche Ltd., Basel, Switzerland) was started. Before the start of the targeted therapy, peripheral blood was collected and circulating tumor cells were isolated in accordance with the protocol. In the peripheral blood sample, 6 CTCs were detected in 9 mL of blood (Figure 5a). Before the third course of targeted therapy with alectinib, 2 CTCs in 9 mL of blood were detected (Figure 5b).

**Figure 6:**
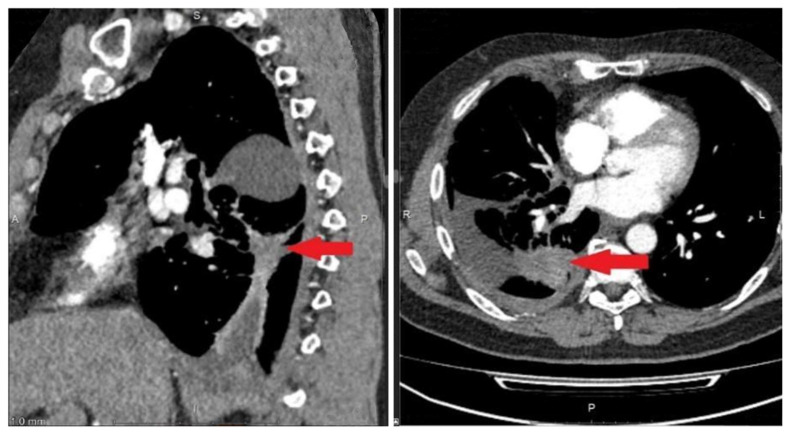
Protocol code 10: CT scan of the patient’s chest dated 19 February 2021. CT signs of fibroatelectasis S6 on the right, right-sided small hydrothorax, in comparison with the previous CT scan dated 17 December 2020, straightening of the lower lobe of the right lung is highlighted, a decrease in the amount of fluid in the right pleural cavity (indicated by arrow).

A control CT scan of the chest was performed and positive dynamics were noted (Figure 6).

**Figure 7:**
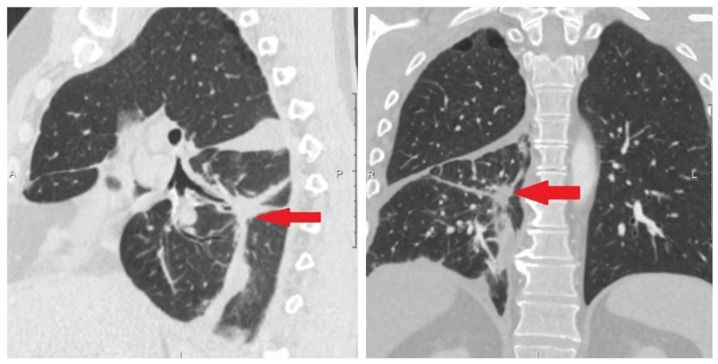
Protocol code 10: CT scan of the chest from 13 May 2021. Fibrous changes at the site of tumor tissue (indicated by arrow).

Before the sixth injection of the targeted drug, no circulating tumor cells were detected in the peripheral blood sample. According to the chest CT data from 3 May 2021, positive curative dynamics was noted in the form of straightening of atelectasis of the lower lobe on the right and a decrease of the amount of fluid in the right pleural cavity (Figure 7).

At the time of analysis (1 January 2024), the patient was alive and continued treatment. The follow-up period from the start of treatment was 39 months. This clinical case demonstrated a decrease in the number of CTCs during ongoing targeted therapy in a patient with a tumor that had a molecular target. A decrease in the number of CTCs during treatment correlates with ongoing partial response according to results of CT.

#### 3.6.2. A Clinical Example Demonstrating a Unfavorable Outcome of the Disease and the Evolution of the Number of CTCs Detected in the Peripheral Blood

**Figure 8:**
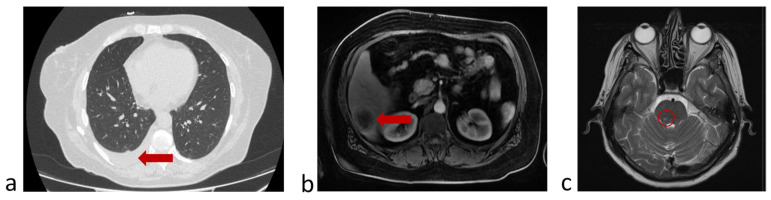
Protocol code 13: (**a**) abdomen MRI from 20 June 2022: the liver structure is heterogeneous due to several (*n* = 3) focal lesions of an oval shape with clear contours, the largest dimensions being 4.1 × 3.1 cm in S6 subcapsular (indicated by arrow); (**b**) chest CT from 26 June 2022 revealed right-sided small hydrothorax (indicated by arrow); (**c**) brain MRI from 24 June 2022: in the substance of the brains on the right, a formation measuring 2 × 5 mm with minimal accumulation of contrast is determined (marked with a circle). All examinations were made before the start of treatment of metastatic cancer.

The 56-year-old female patient, smoker (protocol code 13). She considered herself sick in February 2021, when a cough and fever appeared. In March 2021, she was diagnosed with right-sided lower lobe pneumonia, for which she was treated in a general hospital without effect. Based on the results of the examination (CT scan of the chest, fibrobronchoscopy with biopsy, video thoracoscopy with biopsy) she was diagnosed with cancer of the right lower lobe bronchus T3N2M0, stage IIIB. Histological examination No. 22A1.436 dated 23 March 2021 confirmed a low-differentiated squamous-cell carcinoma G3. A course of radical chemoradiation therapy with a partial response (PR) was carried out (April–May 2021). In a year (June 2022) a disease progression (liver metastases, pleuritis and single brain lesion) was detected (Figure 8) and the patient was included in the study.

**Figure 9:**
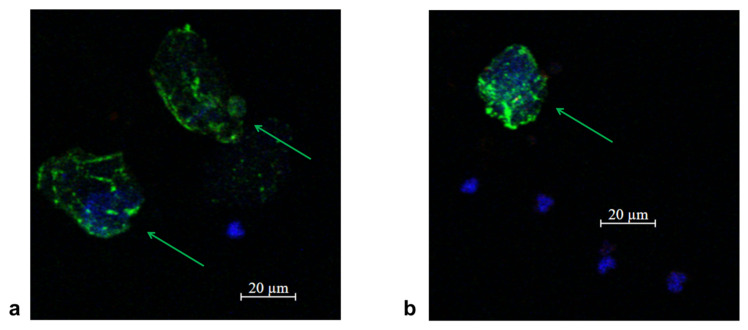
Representative fluorescence confocal microscopy images of tumor cells isolated from the patient’s peripheral blood sample (protocol code 13) at the baseline (**a**) and after 2 treatment courses (**b**). CTC are marked by green arrows in the target sample labelled with CD45 (Red); PCK-26 (Green); DAPI (Blue).

In accordance with the clinical guides of the Russian Association of Oncologists (AOR), taking into account results of histological study (low-grade squamous-cell cancer), immunotherapy with nivolumab (immune checkpoint inhibitor) started 14 July 2022. Before the start of immunotherapy, peripheral blood was collected and circulating tumor cells were isolated in accordance with the protocol. In the peripheral blood sample, 8 CTCs were detected in 9 mL of blood (Figure 9a). After two courses of nivolumab (September 2022), 1 CTCs in 9 mL of blood were detected (Figure 9b). A control chest CT, abdomen MRI and brain MRI demonstrated a stabilization of a disease.

**Figure 10:**
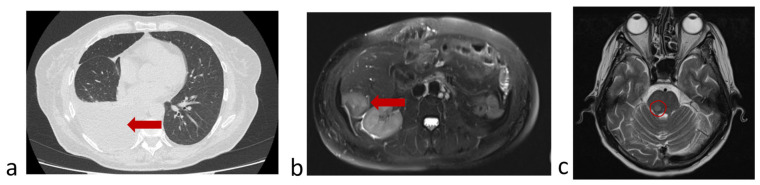
Protocol code 13: (**a**) abdomen MRI from 26 October 2022: the liver structure is heterogeneous due to focal lesions of different sizes (*n* = 6) with unclear uneven contours measuring 4.1 × 3.7 (previously 4.0 × 3.0 cm) with signs of contrast accumulation; (**b**) chest CT from 28 October 2022: Pneumatization of the upper right lobe is reduced. Fluid in the right pleural cavity is up to 64 mm thick (previously 16 mm) with a volume of up to 1750 mL; (**c**) brain MRI from 29 October 2022: in the substance of the brainstem on the right, a formation measuring 7 × 5 mm with accumulation of contrast is determined (marked with a circle).

Before the fourth injection of nivolumab (October 2022), the patient felt worse and disease progression was confirmed by chest CT, abdomen and brain MRI (Figure 10).

The number of CTCs in the peripheral blood at the time of disease progression was 7. After the sixth course of immunotherapy (December 2022), the patient’s condition progressively worsened (ECOG = 3) and further specific treatment was deemed inappropriate. The last blood sample for CTC determination was obtained on 20 December 2022, the number of tumor cells was 7 per 9 mL of blood. The patient then received palliative care and died on 19 March 2023. The overall survival from the first blood sample collection was 8 months.

## 4. Discussion

A non-small cell lung cancer is the most difficult cancer type to analyze using liquid biopsy methods, which has led to conflicting conclusions about the correlation of the therapeutic efficacy with isolated CTC numbers. In our study, a systematic analysis of the correlation of the therapeutic efficacy with the number of CTCs was carried out using the advanced microfluidic technology based on a spiral inertial microfluidic device [34], which provided a useful insight to the existing conflicting results.

Our study confirmed that the CTC separation system based on a spiral inertial microfluidic device allowed identifying tumor cells in the majority of patients with metastatic non-small cell lung cancer. At the start of the treatment, CTCs were detected in 76.6% of the patients, which corresponded to the data obtained using an alternative liquid biopsy technique based on EPCAM-assissted CTC isolation [28]. The initial number of CTCs did not depend on the histological structure of the tumor and the localization of the metastatic process, which was fully consistent with the data of the reported studies of CTCs in lung cancer [23,37,38].

Most studies devoted to the problem of circulating tumor cells in patients with locally advanced and metastatic lung cancer have associated the presence of CTCs before the treatment as an unfavorable prognostic factor correlated with the poor survival [39]. Due to the variability of liquid biopsy techniques and loosely established criteria to discriminate CTCs and non-malignant cells, different cutoff levels from 1 to 8 have been established and led to ambiguous disease prognosis [18,19,20,21,22]. We found that a cut-off level of 3 CTCs/9 mL of blood provided statistically significant discrimination of the patient survival rates, with a risk level of 2.6, in agreement with most studies on the topic.

The most favorable prognosis and high OS were demonstrated in patients with the driver mutations detected in the tumor tissue, and who received appropriate targeted therapy. In contrast to the conclusion reported by Tamminga et al. [24], our study showed that the best survival was demonstrated by patients with tumors in which driver mutations were detected and who received therapy with the corresponding targeted drugs (tyrosine kinase inhibitors), which is consistent with the results of previously conducted clinical trials [40,41]. The number of patients is too small for a robust conclusion, but the obtained results allow “highlighting” indications and limitations of using CTC as a predictive and prognostic marker in patients with metastatic NSCLC.

Overall, the obtained results speak in favor of liquid biopsy of CTC based on the microfluidic system for long-term prognosis of the lung cancer progression and resultant patent survival. The demonstrated liquid biopsy technique is deemed clinically useful for optimization of the treatment regimens by way of periodic monitoring of the presence of CTCs in the blood of individual patients during the treatment.

## 5. Conclusions

The study was carried out using advanced microfluidic technology based on a spiral inertial microfluidic device and provided clarity on the prognostic and predictive value of the baseline CTC count in patients with metastatic lung cancer. It demonstrated that a cutoff level of 3 CTCs/9 mL of peripheral blood has a prognostic value with a HR = 2.6 and a decrease in the number of CTCs after the start of treatment is a criterion for a better response to therapy and longer survival.

## Figures and Tables

**Table 1:** Demographics and clinico-pathological characteristics of study cohort.

Criterion		Number of Patients (%)
Age	41–50	5 (17%)
	51–60	13 (43%)
	61–70	8 (27%)
	>70	4 (13%)
Stage of disease (at time of diagnosis)	IB	1 (3.33%)
	IIA	1 (3.33%)
	IIIa	6 (20%)
	IIIb	1 (3.33%)
	IV	21 (70%)
Histology	Squamous cell carcinoma G2	8 (26.7%)
	Squamous cell carcinoma G3	1 (3.3%)
	Adenocarcinoma G1	2 (7%)
	Adenocarcinoma G2	7 (23.3%)
	Adenocarcinoma G3	12 (39.7%)
IHC markers/presence of mutations	Del 19	3 (10%)
	L858R	2 (6.6%)
	T790M	1 (3.3%)
	ALK	3 (13.3%)
	High PD-L1 expression	7 (23.3%)
	No activating mutations	14 (46.8%)
Drug treatment regimen (1 line)	Target therapy	9 (30%)
	Polychemotherapy	11 (36.7%)
	Polychemotherapy + immunotherapy	5 (17%)
	Polychemotherapy + target therapy	1 (3.3%)
	Immunotherapy	3 (10%)
Changing the line of therapy due to progression	Polychemotherapy—immunotherapy	1 (3.3%)
	Target therapy—immunotherapy—polychemotherapy	1 (3.3%)
Line change after identification of an activating mutation	Polychemotherapy—target therapy	1 (3.3%)
Total		30 (100%)

## Data Availability

Data is contained within Supplementary Materials.

## Supplementary Materials

The following supporting information can be downloaded at: https://www.mdpi.com/article/10.3390/mi16040470/s1, Table S1: Dynamics of the number of circulating tumor cells in patients during drug treatment.

## Aknowlegments

Management and staff of Research Institute of Clinical Oncology (Nizhny Novgorod) for assistance in conducting the research.

## Abbreviations

The following abbreviations are used in this manuscript:

CI	Confidence intervals
CT	Computed tomography
CTC	Circulating tumor cells
ECOG	Eastern Cooperative Oncology Group
EpCAM	Epithelial cell adhesion molecule
HR	hazard ratio
IHC	Immunohistochemistry
MRI	Magnetic resonance imaging
NSCLC	Non-small cell lung cancer
OS	Overall survival
PBS	Phosphate-buffered saline
RBC	Red blood cell
RECIST	Response evaluation criteria in solid tumours
SPECT	Single-photon emission computed tomography
T-sample	Target tube
W-sample	Waste tube
WBC	White blood cell
