# Supplementary material for: Prognostic Value of the Number of Circulating Tumor Cells in Patients with Metastatic Non-Small Cell Lung Cancer
Source: Micromachines (Basel). 2025 Apr 15;16(4):470. doi: 10.3390/mi16040470 (PMC12029726; doi:10.3390/mi16040470)
Supplement: Supplementary file 1 [file micromachines-16-00470-s001.zip › micromachines-3545027-supplementary.pdf]

Table S1. Dynamics of the number of circulating tumor cells in patients during drug treatment.

Patient code	Probe number/Number of detected CTCs in 9 ml blood						Outcome, OS
	1	2	3	4	5	6	
1	7	0	3	2	0	-	Died, 22 months
2	5	3	0	0	3	4	Died, 16 months
3	11	13	-	-	-	-	Died, 3 months
4	10	10	11	-	-	-	Died, 7 months
5	12	-	-	-	-	-	Died, 2.5 months
6	6	4	5	0	0	-	Died, 16 months
7	0	2	0	0	0	0	Alive, 39 months
8	0	0	0	0	0	0	Alive, 39 months
9	7	5	0	4	-	-	Died, 9 months
10	6	2	0	0	0	0	Alive, 39 months
11	4	3	-	-	-	-	Died from causes unrelated to the underlying disease 2.5 months
12	6	0	4	2	-	-	Died, 9 months
13	8	1	5	7	-	-	Died, 8 months
14	16	-	-	-	-	-	Died, 2 months
15	5	5	3	-	-	-	Died, 8 months
16	11	8	0	-	-	-	Died, 7 months
17	5	2	0	0	-	0	Died, 27 months
18	12	14	-	-	-	-	Died, 3 months
19	7	4	0	3	-	-	Died, 7 months
20	0	0	0	0	0	-	Alive, 41 months
21	3	0	3	1	1	-	Died, 29 months
22	0	0	0	6	-	-	Died, 13 months
23	2	0	6	7	-	-	Died, 11 months
24	0	0	1	0	-	-	Died, 26 months
25	8	5	5	7	-	-	Died, 10 months
26	3	2	4	2	-	-	Died, 6,5 months
27	0	0	0	7	7	-	Died, 13 months
28	4	0	0	0	-	-	Died, 22

							months
29	0	7	7	-	-	-	Died, 11,5 months
30	5	11	-	-	-	-	Died, 4 months

The rows highlighted in gray correspond to patients with identified driver mutations in the EGFR and ALK genes who received targeted therapy.
